# *Scedosporium* infection disseminated “from toe to head” in allogeneic stem cell transplant recipient: a case report

**DOI:** 10.1186/s12879-023-08345-2

**Published:** 2023-05-25

**Authors:** Debra A. Marinovic, Eric Bhaimia, Graeme N. Forrest, Rebecca LaRue, Sunita Nathan, Celalettin Ustun, Anna Ward

**Affiliations:** 1 Division of Hematology, Oncology and Cell Therapy, Department of Internal Medicine, Rush University Medical Center, 1725 W Harrison St Suite 809, IL 1725 W Harrison St Suite 809 Chicago, USA; 2 Division of Infectious Disease, Department of Internal Medicine, Rush University Medical Center, Chicago, IL USA; 3 Department of Pharmacy, Rush University Medical Center, Chicago, IL USA

**Keywords:** *Scedosporium*, Allogeneic transplant, Invasive fungal disease, Case report

## Abstract

**Background:**

*Scedosporium* is a lesser-known non-*Aspergillus* genus of mold that can present in unsuspecting ways. If overlooked, it may disseminate and cause high mortality in high-risk allogeneic stem cell transplant recipients.

**Case presentation:**

This case report describes a 65-year-old patient with Acute Myeloid Leukemia who underwent an allogeneic hematopoietic stem cell transplant after a period of prolonged neutropenia with fluconazole prophylaxis. She suffered severe debility with altered mentation from a *S. apiospermum* infection which likely disseminated from a toe wound to the lung and central nervous system. She was successfully treated with liposomal amphotericin B and voriconazole, but faced a prolonged recovery from physical and neurologic sequela.

**Conclusions:**

The case highlights the importance of adequate anti-mold prophylaxis in high-risk patients, and the value of a thorough physical examination in this patient population, with particular attention to skin and soft tissue findings.

## Background

Allogeneic hematopoietic stem cell transplant (alloHCT) offers a potential cure for poor-risk acute myeloid leukemia (AML) and other hematologic malignancies [1]. The need for immunosuppressive therapy, in conjunction with prolonged neutropenia during and often before transplantation, places the recipient at risk for invasive mold infections. Antifungal prophylaxis sometimes does not include anti-mold prophylaxis. As a result, molds like *Aspergillus,* implicated in up to 80% of invasive fungal disease (IFD), can complicate alloHCT [2]. *Scedosporium* is a lesser-known ubiquitous mold that has increasingly been associated with disseminated infections and poor survival [3, 4].

We describe a case of disseminated S*cedosporium* infection in a patient with AML who developed pulmonary and central nervous system (CNS) fungal infection from an unexpected source (right hallux) after alloHCT and was treated successfully. The aim of this case presentation is to reinforce the necessity of adequate anti-mold prophylaxis in patients with prolonged neutropenia prior to transplantation, and to highlight the importance of a thorough “head to toe” physical examination.

## Case

**Fig. 1:**
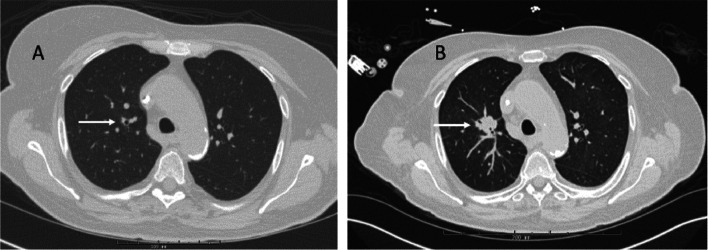
CT chest. Non-contrast computerized tomography (CT) showing right upper lobe nodular opacity 20 days before transplant, measuring 0.4 cm (**A**), and on day + 6 after transplant, measuring 1.6 × 1.8 cm (**B**). The pre-transplant opacities were of indeterminate significance, and attributed to possible resolving infection

The patient is a 65-year-old female who was diagnosed with unfavorable risk AML [with TP53 mutation and del(5q)] in April 2022. She was refractory to standard induction therapy but responded to a combination of a hypomethylating agent and venetoclax (3 cycles). She remained neutropenic throughout and after treatment, and received prophylaxis with fluconazole 400 mg daily for 3 months until her transplant, due to concern for elevated liver enzymes previously with posaconazole. She was neutropenic upon admission for alloHCT [white blood cells (WBC) 0.81 × 10^9^/L and absolute neutrophil count (ANC) 0.62 × 10^9^/L]. Her pre-transplant chest computerized tomography (CT) revealed irregular nodular opacities, therefore posaconazole 300 mg daily was re-challenged on admission (day -6). On day + 5, she was switched to micafungin 150 mg daily with concern for transaminitis. On day + 6, she developed hypoxemia, and had a worsening RUL nodular opacity on chest CT (Fig. 1A, B). Bronchioalveolar lavage (BAL) was performed with negative BAL galactomannan and cultures. Serum β-D-Glucan (Fungitell™ assay) was positive (180 pg/mL, reference range < 60 pg/mL). Her hypoxia resolved with diuresis, and posaconazole was resumed day + 8. On day + 15, neutrophil engraftment was noted and was associated with hypoxia, fevers, and diarrhea. Methylprednisolone (1 mg/kg daily) started on day + 16 for engraftment syndrome led to improvement.

**Fig. 2:**
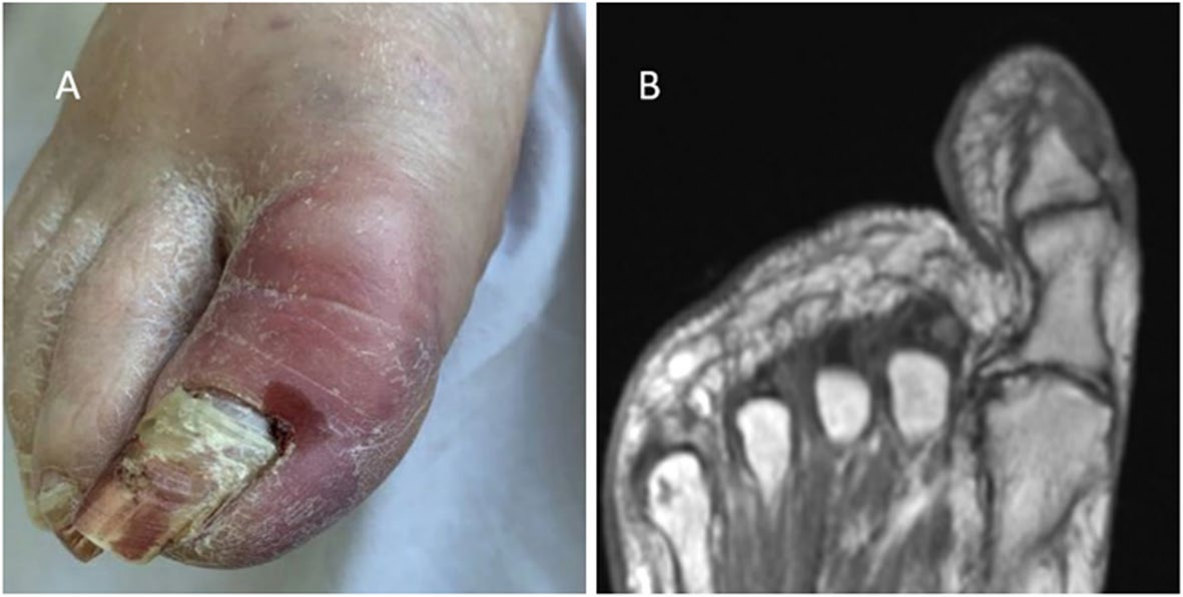
Osteomyelitis. Right Hallux at day + 15 (**A**) and via Magnetic resonance imaging (MRI) day + 54 (**B**). Low T1 signal intensity along the medial distal border of the great toe distal phalanx consistent with osteomyelitis

**Fig. 3:**
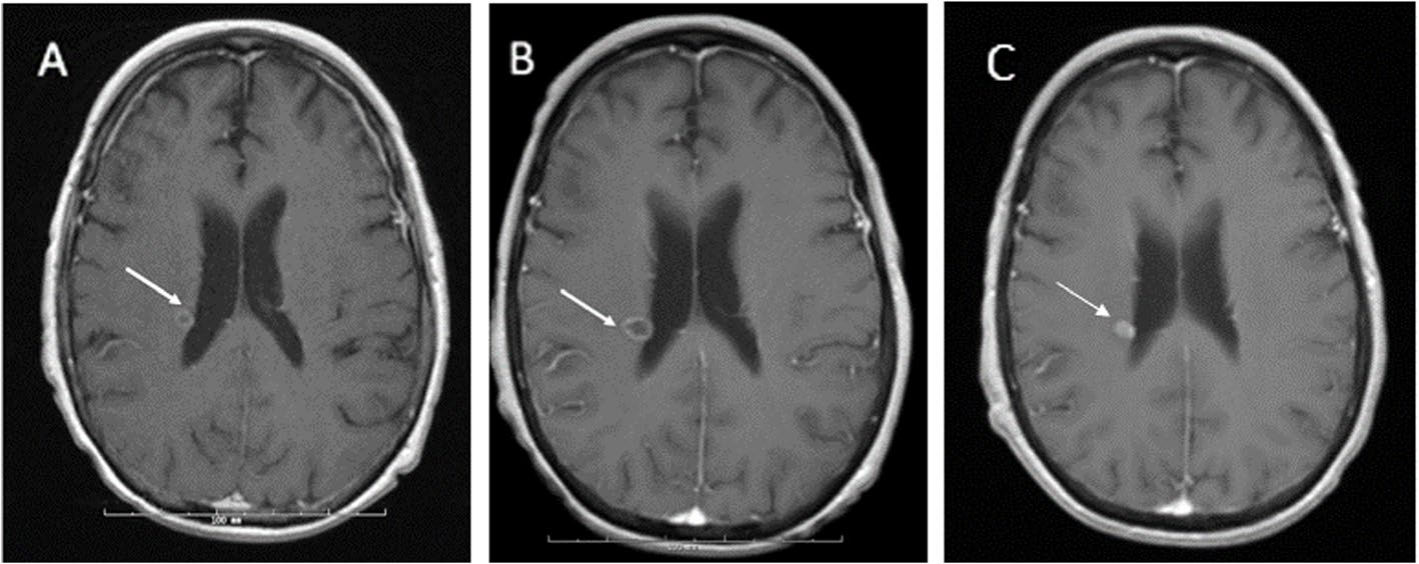
MRI brain. T1 post-contrast imaging with rim-enhancing lesion in periventricular right frontoparietal region, increased from 7 × 4 mm on day + 26 (**A**) to 10 × 7 mm on day + 33 (**B**). After 8 weeks of therapy the lesion decreased back to 7 × 6 mm on day + 83 (**C**)

At the same time, she was noted to have right toe swelling and erythema, initially thought to be traumatic and improved without intervention (Fig. 2A). Onychomycosis was presumed due to the appearance of the nailbed, and topical ketoconazole 2% ointment was applied for the duration of her treatment course. Her performance status declined significantly, and she became more lethargic and less responsive to verbal or non-verbal stimuli. Viral studies including serum human herpesvirus 6 (HHV6), adenovirus, Epstein-Barr virus (EBV), cytomegalovirus (CMV), and blood cultures, were unrevealing. CT of the brain was negative for bleeding or any new abnormality to explain the clinical picture. Psychiatry and neurology evaluations suggested hypoactive delirium and medications were adjusted to account for possible psychogenic side effects. On day + 26, with no improvement in her mental status, brain magnetic resonance imaging (MRI) showed a rim-enhancing lesion in the periventricular right frontoparietal region, concerning for abscess (Fig. 3A). Cerebral spinal fluid (CSF) PCR studies for CMV, Herpes simplex, HHV6, adenovirus, EBV, *Cryptococcus*, and fungal and bacterial cultures were negative, though protein and glucose were elevated. CT chest was suggestive of persistent but improved nodular opacities. Dual antifungal therapy comprising liposomal amphotericin B 3 mg/kg daily and voriconazole 350 mg BID was started. Repeat imaging a week later revealed an increase in size of the abscess with interval new lesions (Fig. 3B). The liposomal amphotericin B dose was increased to 5 mg/kg daily while voriconazole level was therapeutic at 2.8 µg/mL. Her level of alertness waxed and waned; however, she remained bedbound and required enteral nutrition. With no clear improvement, CSF analysis was repeated and remained negative for all pathologic microbiologic agents (repeated as above). Brain biopsy was deferred due to high risk for complications. Her right toe exam was concerning for worsening paronychia. Podiatry noted a positive probe-to-bone test, a finding with greater than 90% positive predictive value for osteomyelitis in high-risk patients, but lower in immunocompetent patients [5]. This finding was further supported by decreased T1 marrow signal intensity on MRI (Fig. 2B), a test with a lower positive predictive value approaching 80% [6]. The probed tissue was cultured, and initially grew coagulase-negative *Staphylococcus* and diptheroids. She received doxycycline 100 mg BID for 4 weeks.

**Fig. 4:**
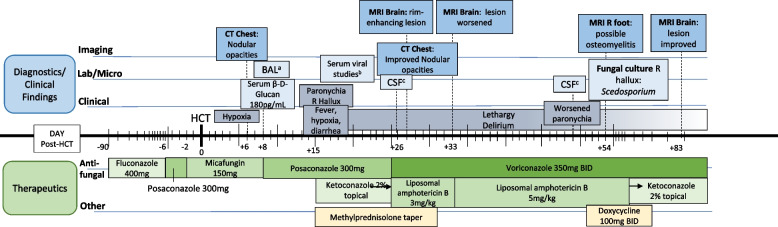
Timeline of clinical course. CT; computerized tomography; MRI, magnetic resonance imaging; BAL, bronchioalveolar lavage; CSF, cerebrospinal fluid ^a^Negative for pneumocystis, galactomannan, listeria, bacterial, AFB, and fungal cultures, and respiratory viral pathogens ^b^Human Herpesvirus (HHV)-6, cytomegalovirus, adenovirus, Epstein-Barr virus (EBV) undetectable ^C^Negative for cytomegalovirus, herpes simplex 1/2, HHV-6, adenovirus, EBV, *cryptococcus*, fungal, AFB, and bacterial cultures. WBC 1/uL, 15 total cells (93% lymphocytes), glucose elevated in the first sample, 82 mg/dL; Protein elevated in both samples, 75.4 mg/dL and 47.4 mg/dL, respectively

After 5 weeks of aggressive antifungal therapy (day + 58), MRI imaging finally showed significant improvement in the brain abscess. On day + 60, fungal cultures from the right hallux grew *Scedosporium apiospermum,* 7 days after specimen collection. Culture media included Sabouraud Dextrose with brain–heart infusion agar, inhibitory mold agar, and brain–heart infusion agar with sheep blood, chloramphenicol, and gentamycin. Laboratory identification was performed at the University of Texas Health Science Center fungus testing laboratory, based on sequencing of internal transcribed spacer, beta-tubulin, and calmodulin loci. Antifungal susceptibilities (using CLSI M38-A2 standard) showed voriconazole minimum inhibitory concentrations (MIC) = 1 µg/ml, posaconazole MIC = 1 µg/mL, amphotericin B MIC = 1 µg/ml and isavuconazole MIC = 8 µg/ml. It should be noted that despite the low MIC, liposomal amphotericin B clinically performs poorly against *Scedosporium* spp. regardless of the MIC range [7]. Liposomal amphotericin B was discontinued and voriconazole continued as monotherapy. At day + 76, her poor performance status remained unchanged despite improvement in her imaging and otherwise excellent organ and graft function, and she was transferred to a rehabilitation facility. A repeat MRI brain on day + 83, after 8 weeks of treatment, showed significant decrease in the size of the lesion (Fig. 3C). Her mental status and cognitive functions eventually returned to baseline during her rehabilitation course. However, she developed failure to thrive due to other non-infectious HCT complications, and opted to transition to comfort care. She ultimately passed away 6 months after her HCT. See Fig. 4 for a timeline of the patient’s clinical course and interventions.

## Discussion

*Scedosporium apiospermum* is one of the more common species of non-*Aspergillus* molds detected in the alloHCT population and is a significant cause of mortality [4]. Our patient possessed several risk factors that predisposed her to this infection. She received a transplant from a matched unrelated donor; did not receive anti-mold prophylaxis despite prolonged neutropenia prior to transplantation, and received high-dose corticosteroid therapy [8]. Moreover, we later learned that her home environment was unsanitary and a possible source for skin inoculation. Disseminated *Scedosporium* spp. can originate from a wide range of local sites [9]; in this case, it possibly began as osteomyelitis of her right hallux despite the classic appearance of paronychia. Several cases of disseminated disease originating from skin/soft tissue infections and osteomyelitis are reported in the literature [10, 11]. Another possibility is inhalation of *S. apiospermum* with secondary dissemination to the brain and soft tissue. *Scedosporium* spp. have low reactivity to the β-D-Glucan assay, but as in this case, a positive test can still occur and may suggest the diagnosis particularly in cases where an invasive fungal infection is suspected but undiagnosed [12]. In this case, surgical sampling of tissue was considered but deferred due to the patient’s high risk of surgical complications. Fungal blood cultures were also negative. Therefore, in the absence of tissue sampling, we cannot prove the diagnosis of a disseminated infection versus a contaminant. However, given the host risk factors, clinical manifestations of lung, brain, and skin involvement, isolation of *Scedosporium* on culture, and serum β-D-Glucan positivity, a diagnosis of disseminated *Scedosporium* is favored, as further supported by continued clinical improvement on voriconazole monotherapy.

Newer triazoles, particularly voriconazole, have been shown to have superior in vitro activity against *S. apiospermum* compared to older triazoles, amphotericin B, and echinocandins [9], and have been associated with better survival in retrospective studies [3]. Isavuconazole does not appear to be as effective as voriconazole. The management of this patient was in line with current guidelines, which are strongly in favor of voriconazole first-line therapy and discourage the use of liposomal amphotericin B [13]. An earlier guideline established by The European Society of Clinical Microbiology and Infectious Diseases [14] recommends surgical debridement in cases of osteomyelitis due to *Scedosporium* spp. (strength of recommendation grade A, quality of evidence level IIr). This option was offered to the patient but she and her family declined in favor of conservative management, which was appropriate given her performance status.

In a systematic review of non-*Aspergillus* mold osteomyelitis cases, the foot was the most commonly involved site (25.3%) [15]. In this review, the median duration of treatment with anti-fungal therapy was 115 days with most patients receiving a combination of surgical debridement and antifungal therapy with good outcomes. There is less data to establish a treatment duration of CNS mold infection. Several months of therapy is likely to be necessary, but the duration of treatment of disseminated scedosporiosis is variable based on clinical response to therapy and net immunosuppression [16].

It is impressive that this high-risk patient survived disseminated *S. apiospermum* infection, including skin, lung, and brain, despite her early post alloHCT status. Many factors contributed to the successful treatment of her IFD, including early and aggressive treatment with effective anti-mold therapy including voriconazole, rapid engraftment with stable high neutrophil counts, tapering of corticosteroids and optimizing immunosuppression as feasible.

This case demonstrates the debilitating and sometimes devastating nature of IFD and highlights the emergence of lesser-known species that can break through common prophylactic antifungals and mimic common infections. The presence of a positive culture, imaging findings consistent with lung and brain involvement, and positive β-D-Glucan all supported the diagnosis of a disseminated *Scedosporium* infection. Although we successfully treated IFD in this patient, the best approach is effective prevention. Moreover, this case demonstrates the importance of a thorough “head-to-toe” physical exam in preventing dissemination from “toe to head.”

## Data Availability

Not applicable.

## Abbreviations

alloHCT: Allogeneic stem cell transplantation
AML: Acute myeloid leukemia
IFD: Invasive fungal disease
CNS: Central nervous system
WBC: White blood cells
ANC: Absolute neutrophil count
CT: Computerized tomography
BAL: Bronchioalveolar lavage
HHV6: Human herpesvirus 6
EBV: Epstein-Barr virus
CMV: Cytomegalovirus
MRI: Magnetic resonance imaging
CSF: Cerebrospinal fluid
BID: Twice daily
MIC: Minimum inhibitory concentration
